# Brush cells fine-tune neurogenic inflammation in the airways

**DOI:** 10.1172/JCI161439

**Published:** 2022-07-01

**Authors:** Qihua Ye, Lora G. Bankova

**Affiliations:** Division of Allergy and Clinical Immunology, Jeff and Penny Vinik Center for Allergic Disease Research, Brigham & Women’s Hospital and Department of Medicine, Harvard Medical School, Boston, Massachusetts, USA.

## Abstract

Airway epithelial cells, once considered a simple barrier layer, are now recognized as providing an active site for antigen sensing and immune response initiation. Most mucosal sites contain chemosensory epithelial cells, rare and specialized cells gaining recognition for their unique functions in sensing and directing the immune response symphony. In this issue of the *JCI*, Hollenhorst, Nandigama, et al. demonstrated that tracheal chemosensory brush cells detected bitter-tasting substances, including quorum-sensing molecules (QSMs) generated by pathogenic *Pseudomonas aeruginosa*. The authors used various techniques, including genetic deletion of brush cells, genetic manipulation of brush cell signaling, deletion of sensory neurons, in vivo imaging, and infection models with *P*. *aeruginosa*, to show that QSMs increased vascular permeability and innate immune cell influx into the trachea. These findings link the recognition of bacterial QSMs to the innate immune response in the airways, with translational implications for airway inflammation and infectious pathology.

## Mucosal immunity and host defense

Chemosensory brush cells are found at nearly all mucosal sites, including the nose, trachea, intestine, gallbladder, urethra, and pancreas, where they are referred to as solitary chemosensory cells (SCCs), brush cells, or tuft cells (1–4). They were identified as chemosensory based on their close interaction with nerve endings in the nose (3) and the trachea (4). This connection was recently verified in healthy human sinonasal mucosa (5). The chemosensing ability of brush cells is defined by the expression of taste receptors and taste transduction machinery (3, 6), in particular the monovalent-selective cation channel, transient receptor potential melastatin 5 (TRPM5) (7, 8). When activated through bitter-taste receptors, chemosensory brush cells in both the nose and the trachea initiate protective respiratory nerve reflexes. Specifically, brush cell–derived acetylcholine and downstream signaling to sensory neurons leads to a respiratory pause (3, 4).

**Figure 1:**
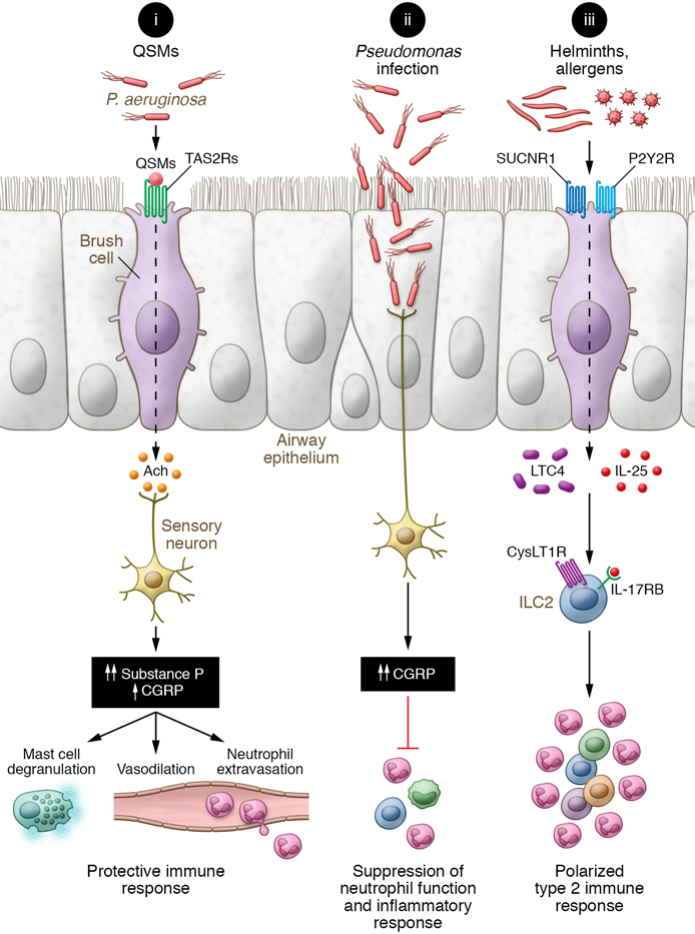
Brush cell–activating receptors direct protective and pathologic responses in the airways. (i) *Pseudomonas* *aeruginosa* colonies communicate with QSMs to increase population growth and virulence. QSMs are detected by bitter-taste TAS2 receptors (TAS2Rs)on brush cells, leading to generation of acetylcholine (Ach), activation of sensory neurons, and release of neuropeptides, predominantly substance P. Substance P induces vascular permeability and neutrophil extravasation and in some instances is associated with mast cell degranulation. (ii) When *P*. *aeruginosa* population size reaches critical numbers, the bacteria directly activate sensory neurons for CGRP release, which is associated with the suppression of neutrophil function and inflammatory response. (iii) Brush cell activation through the SUCNR1 or P2Y2 receptors (P2Y2Rs) elicits the generation and release of IL-25 and cysteinyl leukotrienes, two proinflammatory mediators that synergize to drive a polarized mucosal type 2 immune response. LTC4, leukotriene C4; CysLT1R, cysteinyl leukotriene receptor 1; ILC2, type 2 innate lymphoid cell.

In this issue of the *JCI*, Hollenhorst, Nandigama, et al. uncovered how brush cells direct the mucosal host defense to impending *Pseudomonas* *aeruginosa* infection (9). Brush cells are activated by bitter-tasting substances, including several quorum-sensing molecules (QSMs) from *P*. *aeruginosa*. QSMs are signal molecules released and perceived by bacteria to coordinate cooperative behavior such as increasing population size and virulence (10). Brush cell recognition of the prototypical bitter-tasting ligand denatonium or QSMs from *P*. *aeruginosa* led to TRPM5- and substance P–dependent increases in vascular permeability and neutrophil influx. Interestingly, the authors also found that higher doses of bitter-tasting ligands triggered increased vascular permeability and neutrophil extravasation independent of brush cells. Deletion of TRPA1-expressing sensory neurons ablated both the brush cell–dependent and brush cell–independent changes in permeability and neutrophil influx, suggesting that sensory neurons are required to detect both low- and high-dose bitter-tasting QSMs. This finding suggests that brush cells augment the ability of sensory neurons to detect the presence of bacteria even before they reach critical numbers for infection by responding to the bacterial communication signals (Figure 1). As bacteria such as *P*. *aeruginosa* use QSMs to communicate and increase numbers and virulence, the brush cell recognition system might serve as a sensor of impending bacterial invasion. Consistently, the authors demonstrated decreased lung inflammation and improved survival when bitter-taste signaling was disrupted in mice infected with very low-dose *P*. *aeruginosa* (9).

## Fine-tuning the activation of sensory neurons

In the nose, activation of SCCs (the nasal counterpart of brush cells) through bitter-taste receptors leads to similar sensory nerve– and substance P–dependent plasma extravasation (11). This sensory nerve activation is associated with the degranulation of mast cells (11). Sensory nerve activation and substance P release in the mouse footpad similarly leads to neutrophil extravasation and mast cell degranulation (12). Hollenhorst, Nandigama, et al. found no evidence of mast cell degranulation in the trachea, which might be explained by the tissue-specific expression of activating receptors in mast cells, or it might be due to differences in innervation of nasal and tracheal mast cells (13).

Previously, Baral et al. showed that activation of sensory neurons suppresses neutrophil influx to the lung in response to *Staphylococcus aureus* infection (14). Deleting sensory neurons improves survival from *S*. *aureus*–induced pneumonia and promotes neutrophil influx through a calcitonin gene–related peptide–dependent (CGRP-dependent) mechanism (14). Similarly, *P*. *aeruginosa* directly activates nociceptors and induces CGRP release, suppressing neutrophil extravasation in murine corneas (15). In contrast, Hollenhorst, Nandigama, et al. found that nociceptor activation through brush cells promotes neutrophil extravasation, vascular permeability, and vasodilatation, and attribute these properties to the combined effect of sensory nerve–derived substance P and CGRP. The experiments clearly demonstrated that mice lacking substance P generation have reduced neutrophil influx and vascular permeability, in agreement with other studies (12). However, the exact source and role of CGRP in this QSM-driven model of brush cell activation remain unclear. Single-cell analyses show that CGRP and substance P are rarely found coexpressed in sensory neurons, and other potentially relevant sources of CGRP were not addressed here. Thus, it remains to be clarified how the interaction of brush cells and sensory neurons directs responses to bacterial invasion. It is likely that brush cells are poised to sense the QSMs that bacteria use to coordinate tissue invasion. This attribute would allow brush cells to direct the early response and expel the bacteria before they reach a quorum. Once bacteria reach critical numbers, they would activate sensory neurons directly to suppress the immune response (Figure 1).

## Paracrine secretion and the response to specific stimuli

Interestingly, Hollenhorst, Nandigama, et al. also found that bitter-taste receptor ligands trigger the release and accumulation of endopeptidases and complement components in the subepithelial compartments of tracheas (9). Incubation of *P*. *aeruginosa* cultures with these tracheal supernatants had a bacteriostatic effect. How brush cells direct the accumulation of these substances remains to be clarified. Notably, bitter-taste receptor activation of nasal SCCs in cultures or explants leads to rapid secretion of antimicrobial peptides from neighboring epithelial cells (16). Thus, paracrine secretion is also a likely contributing mechanism.

It is intriguing to speculate on the activating signals and receptors that direct brush cells to initiate protective responses to expel bacteria or to promote chronic type 2 inflammation. The findings in Hollenhorst, Nandigama, et al. add to the growing body of literature indicating that the activation of taste receptors on tracheal brush cells leads to acetylcholine release, which promotes most of the protective brush cell-initiated responses, including increased mucociliary clearance, respiratory reflexes, and vascular permeability with extravasation of neutrophils (4, 9, 17).

Additionally, brush cells are a dominant epithelial source of IL-25 and a key initiator of type 2 inflammation in the intestine (1, 18, 19) and in the airways (20, 21). Brush cells from all mucosal sites also express a core set of enzymatic cascade components for cysteinyl leukotriene and prostaglandin D_2_ generation, both potent proinflammatory mediators (21–23). Brush cell sensing of allergens in the airways engages an ATP-dependent feed-forward loop through the purinergic P2Y2 receptor for cysteinyl leukotriene generation (21). In the intestine, sensing of helminth products through the succinate receptor (SUCNR1) induces cysteinyl leukotriene– and IL-25–dependent activation of innate type 2 lymphoid cells (24). The synergy of cysteinyl leukotrienes and IL-25 generated by brush cells in response to allergens and helminths promotes a strongly polarized type 2 immune response in both the airways and the intestine (21, 24) (Figure 1).

A distinct brush cell pathway recognizes bacterial components in the gallbladder through free fatty acid receptor 2 (FFAR2). Subsequent signaling leads to the generation of acetylcholine and cysteinyl leukotrienes that independently promote bacterial clearance (25). In the intestine, brush cells generate PGD_2_ to promote mucin release and antimicrobial responses when activated via the vomeronasal receptor Vmn2r26 (26). In addition, brush cells’ effects on the immune system are finely tuned by the tissue environment. O’Leary et al. found that in the absence of brush cells, neutrophil counts are elevated in the gallbladder but unchanged in the spleen, small intestine, and peripheral blood (2).

Altogether, brush cells are poised to respond to bacterial metabolites, stress signals, allergens, and helminths, by engaging a variety of receptors. They either expel pathogens mechanically and promote innate immune cell influx or initiate type 2 inflammation when they sense helminths or allergens (Figure 1).

## Conclusions and clinical implications

Hollenhorst, Nandigama, et al. (9) demonstrated that tracheal brush cells detected bacterial metabolites, especially those that indicate an impending increase in bacterial load. Brush cells then initiate sensory nerve activation with neuropeptide release to direct innate immune cell influx. Thus, brush cells can augment the functions of sensory neurons to detect the presence of bacteria while amplifying the overall antimicrobial host defense. Collectively, the airway chemosensory cells use a wide armamentarium to fight off impending bacterial invasion — antimicrobial and neuropeptide secretion, mast cell degranulation, innate immune cell recruitment, and respiratory reflex activation — to rapidly attack and expel bacteria at the point of entry in the airways.

## Version 1 07/01/2022

Electronic publication
